# Genome-Wide Profiling of Histone H3 Lysine 4 and Lysine 27 Trimethylation Reveals an Epigenetic Signature in Prostate Carcinogenesis

**DOI:** 10.1371/journal.pone.0004687

**Published:** 2009-03-05

**Authors:** Xi-Song Ke, Yi Qu, Kari Rostad, Wen-Cheng Li, Biaoyang Lin, Ole Johan Halvorsen, Svein A. Haukaas, Inge Jonassen, Kjell Petersen, Naomi Goldfinger, Varda Rotter, Lars A. Akslen, Anne M. Oyan, Karl-Henning Kalland

**Affiliations:** 1 The Gade Institute, University of Bergen, Bergen, Norway; 2 Department of Urology, Union Hospital, Tongji Medical College, Huazhong University of Science and Technology, Wuhan, People's Republic of China; 3 Swedish Medical Center, Seattle, Washington, United States of America; 4 Zhejiang-California International Nanosystems Institute (ZCNI), Zhejiang University, Hangzhou, People's Republic of China; 5 Department of Pathology, Haukeland University Hospital, Bergen, Norway; 6 Department of Surgical Sciences, University of Bergen, Bergen, Norway; 7 Department of Surgery, Haukeland University Hospital, Bergen, Norway; 8 Computational Biology Unit, Bergen Center for Computational Science, University of Bergen, Bergen, Norway; 9 Department of Informatics, University of Bergen, Bergen, Norway; 10 Department of Molecular Cell Biology, Weizmann Institute of Science, Rehovot, Israel; 11 Department of Microbiology and Immunology, Haukeland University Hospital, Bergen, Norway; Ordway Research Institute, United States of America

## Abstract

**Background:**

Increasing evidence implicates the critical roles of epigenetic regulation in cancer. Very recent reports indicate that global gene silencing in cancer is associated with specific epigenetic modifications. However, the relationship between epigenetic switches and more dynamic patterns of gene activation and repression has remained largely unknown.

**Methodology/Principal Findings:**

Genome-wide profiling of the trimethylation of histone H3 lysine 4 (H3K4me3) and lysine 27 (H3K27me3) was performed using chromatin immunoprecipitation coupled with whole genome promoter microarray (ChIP-chip) techniques. Comparison of the ChIP-chip data and microarray gene expression data revealed that loss and/or gain of H3K4me3 and/or H3K27me3 were strongly associated with differential gene expression, including microRNA expression, between prostate cancer and primary cells. The most common switches were gain or loss of H3K27me3 coupled with low effect on gene expression. The least prevalent switches were between H3K4me3 and H3K27me3 coupled with much higher fractions of activated and silenced genes. Promoter patterns of H3K4me3 and H3K27me3 corresponded strongly with coordinated expression changes of regulatory gene modules, such as *HOX* and microRNA genes, and structural gene modules, such as desmosome and gap junction genes. A number of epigenetically switched oncogenes and tumor suppressor genes were found overexpressed and underexpressed accordingly in prostate cancer cells.

**Conclusions/Significance:**

This work offers a dynamic picture of epigenetic switches in carcinogenesis and contributes to an overall understanding of coordinated regulation of gene expression in cancer. Our data indicate an H3K4me3/H3K27me3 epigenetic signature of prostate carcinogenesis.

## Introduction

Epigenetics refers to heritable, but potentially reversible, alternated phenotypic states without difference in genotype. The proteins that mediate epigenetic changes are involved in dynamic transcriptional control of gene expression and are encoded by more than 100 genes including DNA methyltransferases (*DNMT*s), histone acetyltransferases (*HAT*s), histone deacetylases (*HDAC*s), histone methyltransferases (*HMT*s), histone demethylases (*HDMT*s) and chromatin remodelling enzymes [1]. EZH2 (Enhancer of Zeste homolog 2), a known core component of the polycomb repressive complex 2 (PRC2), is one of the best characterized HMTs, and can trimethylate the histone H3 lysine 27 (H3K27) and thereby repress gene transcription [2]. Previous work has shown that EZH2 was significantly upregulated and associated with high proliferation rate and aggressive tumor subgroups in prostate cancer [3]. However, the targets and output of epigenetic regulation in prostate cancer are still not completely understood.

Genome-wide profiling of the H3K27me3 modification in prostate cancer has been carried out by a few groups. Yu *et al.* have analyzed H3K27me3 location and suggested a polycomb repression signature in metastatic prostate cancer [4]. It would be informative if H3K27me3 locations were also mapped in benign tissues to show the cancer specificity of the signature. Very recently, H3K27me3 modifications were mapped in both prostate cancer and normal cell lines and a set of genes silenced by EZH2-mediated H3K27 trimethylation specifically in prostate cancer was identified [5]. Both works shed light on the silencing function of EZH2 in prostate cancer, but little is known about epigenetic gene activation in prostate carcinogenesis.

To systematically examine the role of epigenetic regulation in prostate cancer, we have screened dysregulated genes in prostate cancer tissues and cell lines using microarray techniques. We found that the most significantly changed epigenetic regulators in both prostate cancer tissues and cell lines were *EZH2*, *SMYD3* and *DNMT3A*, which function as H3K27 trimethyltransferase, H3K4 di/tri-methyltransferase [6] and DNA methyltransferase [7], respectively. Trimethylation of H3K27 (H3K27me3) and trimethylation of H3K4 (H3K4me3) are associated with repression and activation of gene transcription, respectively [2], [6]. To examine the hypothesis that dysregulated genes in prostate cancer contain a distinct pattern of H3K4me3 and H3K27me3, ChIP-chip analysis was performed for genome-wide profiling of H3K4me3 and H3K27me3 modification patterns in both prostate primary cells and cancer cells. Comparison of the epigenetic switches and gene expression switches between normal primary and cancer cells indicated an H3K4me3/H3K27me3 epigenetic signature in prostate carcinogenesis.

## Results

### Dysregulated Epigenetic Genes in Prostate Cancer

**Figure 1:**
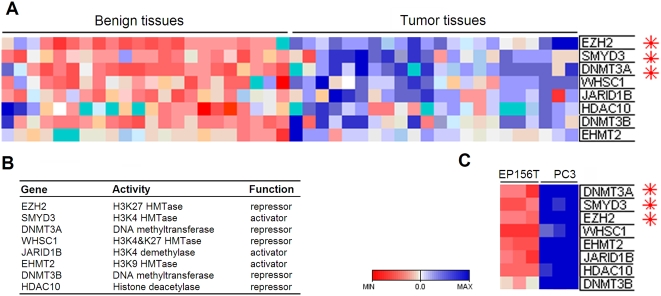
Microarray-based Screening of Changed Epigenetic Genes in Prostate Cancer. A. The top significantly changed epigenetic genes in prostate cancer were overexpressed *EZH2*, *SMYD3* and *DNMT3A*. B. The most significantly changed epigenetic genes in prostate cancer were involved in histone 3 lysine methylation. C. Changed epigenetic genes in prostate cancer tissues have the same changed patterns as the prostate cancer cell line PC3 compared with primary prostate EP156T cells. Asterisks indicate gene expressions with fold change values>1.5 and p-values<3E-10 in prostate cancer tissues compared with benign tissues.

To analyze the dysregulated epigenetic genes in prostate cancer, we screened the microarray gene expression data of prostate cancer and benign tissues as previously published [8], [9] and created a subset of the summarized epigenetic genes, and ranked them according to fold change between prostate benign and cancer tissues. The top three (fold change>1.5, p-value<3E-10) changed epigenetic enzymes were *EZH2*, *SMYD3* and *DNMT3A* (Figure 1A, Table S1 in the Supplemental Data available online). All of them were overexpressed in prostate cancer. EZH2 is a known H3K27 trimethyltransferase, catalyzing H3K27me3, a transcriptional repressive mark [2]. SMYD3 is an HMTase specific for both di- and trimethylation of H3K4, catalyzing H3K4me2/me3, a transcriptional activating mark [6]. DNMT3A is a *de novo* DNA methyltransferase repressing gene transcription [7]. To our knowledge, this is the first report of overexpression of both *SMYD3* and *DNMT3A* in prostate cancer tissues. Very interestingly, five of the top eight changed epigenetic genes were involved in histone H3 lysine methylation (Figure 1B).

We also did genome-wide profiling of the expression patterns of prostate cell lines and screened the changed epigenetic genes between them using the same strategy as in prostate tissues. PC3 cells and EP156T cells were used as prostate cancer and primary cells, respectively. The PC3 cell line was initiated from a bone metastasis of a prostatic adenocarcinoma. The EP156T cell line was established from benign prostate tissue and was characterized as an immortalized primary prostate epithelial cell line [10]. As shown in Figure 1C, the top eight differentially expressed epigenetic genes in prostate cancer have the same expression patterns as in PC3 cells compared with EP156T cells. The high consistency of the most significantly changed epigenetic genes between prostate tissues and cell lines suggested that main epigenetic regulations in prostate carcinogenesis could be mediated by histone H3 lysine methylations.

### Genome-wide Analysis of H3K4me3 and H3K27me3 Modifications in Prostate Cells

It was very interesting to identify *EZH2* and *SMYD3* as the most significantly changed and overexpressed histone modifiers in prostate cancer, since the two modifications are antagonistic and correspond to repressive (H3K27me3) and active (H3K4me3) gene transcription, respectively. To examine the epigenetic signature of H27K4me3 and H3K4me3 in prostate cancer, chromatin immunoprecipitation (ChIP) coupled with promoter microarray (chip) analysis was performed for genome-wide profiling of H3K4me3 and H3K27me3 modification patterns. The prostate cancer cell line PC3 and primary epithelial EP156T cells were selected for profiling because the changed epigenetic gene expression patterns between them were similar to prostate cancer vs benign tissues *in vivo* (Figure 1). DNA fragments pulled down by ChIP-grade antibodies against H3K4me3 and H3K27me3 were hybridized to Agilent human promoter microarrays which comprise ∼244,000 60-mer oligonucleotide probes spaced every ∼195 bp across promoter regions including −5.5 Kb upstream and +2.5 Kb downstream of identified transcriptional start sites (TSS). The probes covered ∼17,000 of the best-defined human transcripts represented as RefSeq.

**Figure 2:**
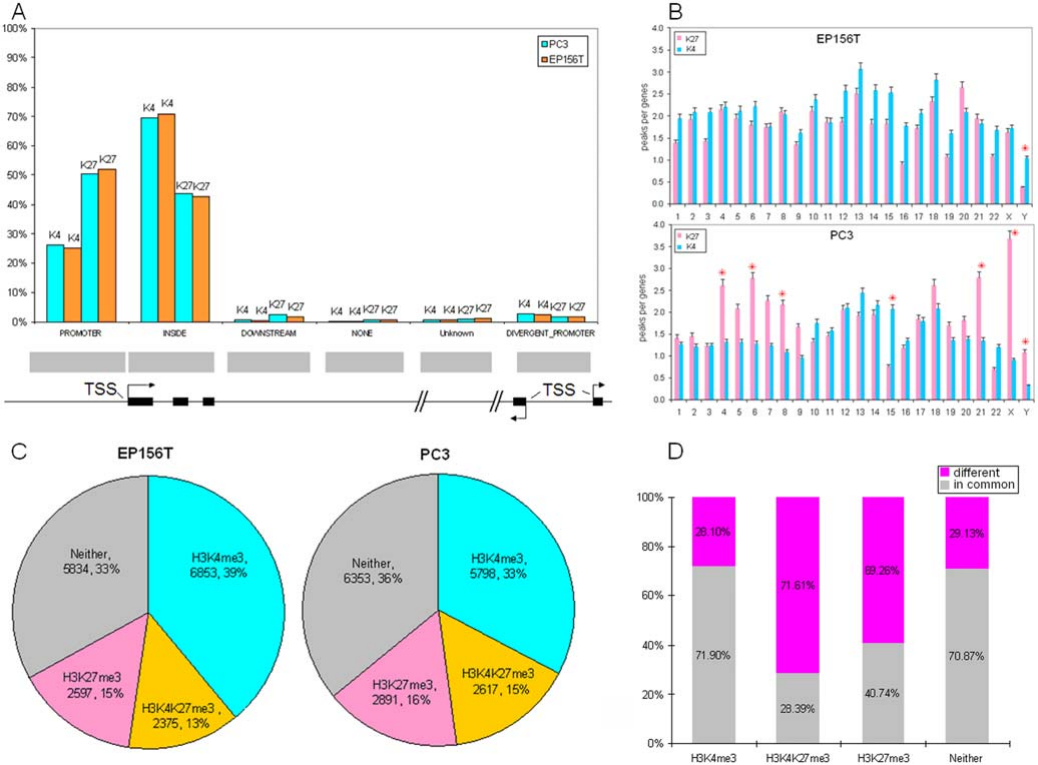
Whole-Genome Distributions of H3K4me3 and H3K27me3 in Prostate Cells. A. Distribution of H3K4me3 and H3K27me3 peaks related to transcription start site (TSS) and the following subregions: PROMOTER (−5.5 Kb upstream from the TSS), DIVERGENT PROMOTER (upstream from two genes that are transcribed in opposite directions), INSIDE (inside a gene), DOWNSTREAM (+2.5 Kb beyond the end of gene), NONE (no genomic features upstream or downstream of the probe) and UNKNOWN (no known information regarding presence of genomic features upstream or downstream of the probe). B. Distribution of H3K4me3 and H3K27me3 peaks in chromosomes, asterisks show chromosomes which differ more than twofold in peak densities of H3K4me3 and H3K27me3. C. Percentages of H3K4me3 only, H3K27me3 only, bivalent H3K4&K27me3 and Neither marked genes in PC3 and EP156T cells. D. Numbers and percentages of genes that are different and in common between PC3 and EP156T within the different categories of H3K4me3 and H3K27me3 mark combinations.

More H3K27me3 peaks (36922) than H3K4me3 peaks (28837) were identified in PC3 cells, while more H3K4me3 peaks (41211) than H3K27me3 peaks (34480) were found in EP156T cells (Table S2, S3, S4, and S5). A similar higher abundance of H3K27me3 marks in PC3 compared to normal cells was very recently reported by another group [11], suggesting that the polycomb system is globally more active in PC3 cells. These peaks can be divided into 6 categories according to probe location in relation to the nearby TSS (Figure 2A). In both cell lines, most H3K4me3 peaks accumulated inside RefSeq genes, while most H3K27me3 peaks mapped to promoter regions (Figure 2A).

In PC3 cells the identified H3K4me3 and H3K27me3 peaks corresponded to 8415 and 5508 genes, respectively. The numbers were 9228 and 4972 genes in EP156T cells, respectively. Calculations of peaks per gene suggested more extensive H3K27me3 than H3K4me3 modifications of both cell lines, especially in PC3 cells (6.7 peaks/gene vs 3.4 peaks/gene), and comparable to human embryonic stem (ES) cells [12], [13]. To confirm the histone H3 trimethylations identified by ChIP-chip, ChIP-qPCR was used to detect selected genes in both cell lines with high correlation between the two methods (Figure S1). We compared 200 H3K27me3 marked genes in PC3 cells reported recently [5] to our promoter array data, and found that 158 genes have the same gene symbols in our Agilent file. About 70% (111 out of 158) overlapped with H3K27me3 marked genes in our data, supporting reliable data on both sides.

To compare peak distribution in individual chromosomes, we normalized the chromosome peak densities by gene numbers in each chromosome. As shown in Figure 2B, most chromosomes except Chr Y have similar peak densities of both H3K4me3 and H3K27me3 in EP156T cells, while it is quite different in PC3 cells. Many chromosomes have much higher (more than two fold) peak densities of H3K27me3 than of H3K4me3, including Chr 4, 6, 8, Y and especially Chr X, while Chr 15 has many more H3K4me3 peaks. It is very interesting that most genes (84%, 439/572) of the X chromosome of PC3 cells were modified by H3K27me3 (Table S6), since it was reported that global enrichment of H3K27me3 in the X chromosome was involved in early steps of X-chromosome inactivation in ES cells [14], [15]. The Y chromosome is also interesting because it has many more H3K4me3 peaks in EP156T in contrast to more H3K27me3 in PC3 cells.

### Bivalent H3K4me3 and H3K27me3 Modified Genes were Strikingly Different in Prostate Primary and Cancer Cells

Bernstein *et al.* surveyed H3K4me3 and H3K27me3 in 60.3 Mb of murine ES cell genome and uncovered “bivalent domains” that show promoters harboring both H3K4me3 and H3K27me3 modified sites [12]. The bivalent modification was proposed to keep the key developmental genes in poised states for later activation. However, recent genome-wide mapping of histone modifications indicates that bivalent-modified domains are not only unique to ES cells but also can be found in differentiated cells such as T cells and mouse embryonic fibroblasts (MEFs) [16], [17], [18].

According to the H3K4me3 and H3K27me3 modification patterns, all genes in PC3 and EP156T cells were divided into four categories: 1) H3K4me3 only, 2) H3K27me3 only, 3) bivalent H3K4&K27me3 and 4) neither H3K4me3 nor H3K27me3 (Neither) (Table S7, 8). The distributions were quite similar in both cells (Figure 2C): around one-third of genes were associated with H3K4me3 only, one-sixth of genes with H3K27me3 only and one-sixth with bivalent H3K4&K27me3. Around half of all H3K27me3 modified genes were also H3K4me3 modified in both PC3 and EP156T cells, showing that bivalent H3K4me3 and H3K27me3 can be found in both prostate somatic cells and cancer cells. However, a comparison of genes within each group revealed striking differences between EP156T cells and PC3 cells. The majority (71.6%) of bivalently H3K4&K27me3 and H3K27me3 only (69.3%) marked genes differed between EP156T and PC3, while only minor proportions of genes were different within the H3K4me3 and Neither groups (Figure 2D). This suggested that epigenetic reprogramming of bivalent H3K4&K27me3 and H3K27me3 marks happened from primary prostate cells to cancer cells, but the extent of the two modifications was kept at similar levels in both cells.

### Correlation between H3K4me3 and H3K27me3 modifications and gene expression

**Figure 3:**
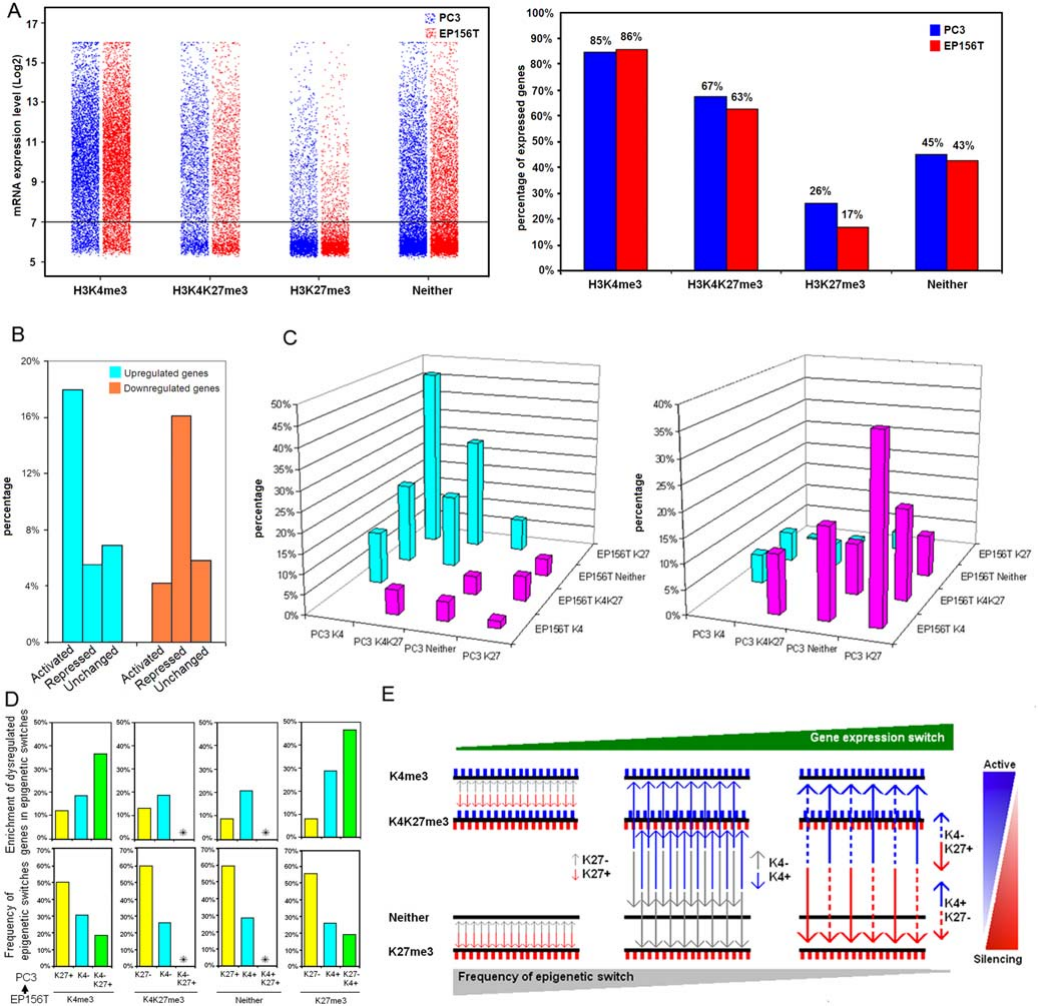
Correlation of H3K4me3 or H3K27me3 Marks with Gene Expression Levels in Prostate Cells. A. Expression levels of genes with different epigenetic marks. The expression levels of individual genes were plotted (left). Percentages of expressed genes in each category are shown (right) with the threshold of mRNA expression set to 7 in J-Express. B. Enrichment of up- or downregulated genes in PC3 compared with EP156T cells within epigenetically repressed, activated and unchanged groups. C. Enrichment of up- (left) or downregulated (right) genes in PC3 compared with EP156T cells in different epigenetic changes. Blue means epigenetically activated and red means epigenetically repressed changes from EP156T to PC3. D. The frequency of epigenetic switches as indicated from EP156T cells to PC3 cells (arrow) shown below and the corresponding enrichment of dysregulated genes in PC3 compared with EP156T cells shown above. Asterisks indicate that switches between Neither and bivalent K4K27me3 were omitted because of too few overlapping genes and low enrichment of dysregulated genes. E. Schematic model to show inverse relationship between the frequency of different combinations of epigenetic switches in primary and cancer cells and the enrichment of dysregulated genes in the different categories of epigenetic changes as depicted graphically in D.

The mRNA expression levels of H3K4me3 and H3K27me3 marked genes in PC3 and EP156T cells were analyzed based on microarray data. Gene expression levels were compared between four groups. As shown in Figure 3A, very consistent relationships were found in both cell lines. H3K4me3 only genes were highest expressed, followed by the bivalent H3K4&K27me3 and Neither genes. The least expressed genes were associated with H3K27me3 only. This suggests that loss of H3K4me3 (change to Neither or H3K27me3 only) or gain of H3K27me3 (change to bivalent H3K4&K27me3 or H3K27me3 only) will repress gene expression. Loss of H3K27me3 (change to Neither or H3K4me3 only) or gain of H3K4me3 (change to bivalent H3K4&K27me3 or H3K4me3 only) will enhance gene expression. The rank of gene expression levels among these four groups in prostate cells shows close agreement with ES cells [13], [17], [19]. However, the bivalently marked genes were rather active and only moderately less expressed than H3K4me3 only marked genes in both EP156T and PC3 cells (Figure 3A).

To investigate the relationship between changed H3K4me3 and H3K27me3 modification patterns and differentially expressed genes in PC3 cells compared with EP156T cells, three subsets were created based on epigenetic changes of H3K4me3 and H3K27me3: 1) repressed: gene loss of H3K4me3 and/or gain of H3K27me3; 2) activated: loss of H3K27me3 and/or gain of H3K4me3; 3) unchanged (Figure 3B). There were many more repressed genes (4134) than activated genes (2932) according this standard (Table S9). Comparing upregulated and downregulated genes in PC3 cells (Table S10) with the three subsets, we found that upregulated genes were enriched significantly in the activated subset, while downregulated genes were enriched significantly in the repressed subset (Chi-square test, p<0.01) (Figure 3B). Our data indicate that loss or gain of H3K4me3 and H3K27me3 is strongly associated with differential gene expression between prostate cancer cells and primary cells.

We further asked which epigenetic pattern changes that mainly contribute to the up- and downregulation from primary to cancer prostate cells. There are 6 combinations of both repressive and activating changes of H3K4me3 and H3K27me3 modifications. For repressive changes: H3K4me3 to H3K27me3 or H3K4&K27me3 or Neither; H3K4&K27me3 to Neither or H3K27me3; Neither to H3K27me3; for activating changes: H3K27me3 to H3K4me3 or H3K4&K27me3 or Neither; Neither to H3K4&K27me3 or H3K4me3; H3K4&K27me3 to H3K4me3. A comparison of gene expression among these combinations showed that the highest enrichment of differentially expressed genes was associated with the switches between H3K27me3 and H3K4me3. Up to 46.6% of H3K27me3 to H3K4me3 genes were upregulated in PC3 cells with extremely low overlap with downregulated genes (0.37%), and 36.9% of H3K4me3 to H3K27me3 genes were downregulated in PC3 cells with extremely low overlap with upregulated genes (1.8%) (Chi-square test, p<0.01), while the lowest enrichment of differentially expressed genes belonged to the switches between H3K27me3 only & Neither and H3K4me3 only and H3K4&K27me3 (from 8% to 13%) (Figure 3C). This is consistent with the notion that both H3K27me3 only and Neither are transcriptional silencing marks, and H3K4me3 only and H3K4&K27me3 are both activating marks. These findings indicate that the switches between H3K27me3 only and H3K4me3 only play the most important role in differential gene expression between prostate normal and cancer cells.

A very interesting inverse relationship was observed between enrichment of dysregulated genes and the frequency of the six epigenetic switches in normal and cancer cells. The most frequent switches were associated with the lowest enrichment of up- or downregulated genes between the two cells. The least frequent switches have the highest enrichment of dysregulated genes (Figure 3D). The most frequent switches were between H3K4me3 and H3K4&K27me3 and between H3K27me3 and Neither. Both switches included either gain or loss of H3K27me3 (H3K27me3+/−), and were associated with low enrichment of changed gene expressions. An intermediate number of epigenetic switches took place between H3K4me3 and Neither and between H3K4&K27me3 and H3K27me3. Both were involved in gain or loss of H3K4me3 (H3K4me3+/−), but affected gene expression intermediately. The least prevalent epigenetic switches occurred between H3K4me3 and H3K27me3, which were involved in H3K27me3+ & H3K4me3− or H3K4me3+ & H3K27me3−, but were associated with the highest percentage of genes that changed expression. This observation suggested that, from normal cells to cancer cells, the most numerous epigenetic changes were either gain or loss of H3K27me3, but this was associated with the lowest propensity to gene expression changes. The least numerous switches occurred between H3K4me3 and H3K27me3 but most frequently resulted in gene expression changes (Figure 3E). The interpretation is that the epigenetic marks under study are strongly linked to gene expression changes, but gain or loss of one single marker is usually not sufficient for transcriptional changes. Instead, combinations of changed epigenetic marks, which occur less frequently than changes of single marks, seem necessary in order to achieve threshold values that lead to efficient silencing or activation of genes, possibly by precipitating cascade reactions.

### Genes with Different Epigenetic Modification Patterns were Associated with Distinct Biological Functions

**Figure 4:**
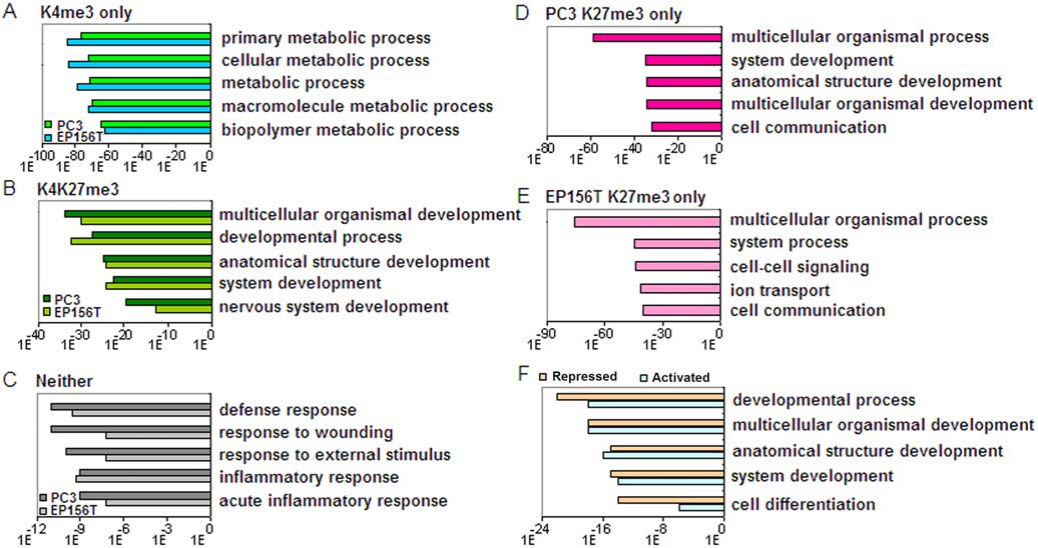
Gene Ontology (GO) Analysis of Genes Associated with Different Histone Modification Patterns. The y axis shows the GO term. The x axis shows the p-value concerning significant enrichment of the top 5 GO terms.

Gene ontology analysis of genes with different epigenetic modification patterns showed distinct biological functions in EP156T and PC3. Most interestingly, the top 5 enriched terms of H3K4me3 only, H3K4H27me3 and Neither groups in both cell lines were exactly the same. As shown in Figure 4A, B, C most of the enriched genes associated with H3K4me3 only were involved in cellular metabolic process, suggesting that genes necessary to cell basic physical functions were active in both cell lines, while genes with neither modification showed very low enrichment values and rather were involved in cell response functions. It is notable that genes with the bivalent H3K4&K27me3 modification were highly related to developmental functions in both cell lines, and the bivalent H3K4&K27me3 group contained the highest fraction of different genes when primary and cancer cells were compared (Figure 2D). Concerning genes associated with H3K27me3 only, the most enriched terms in PC3 cells were involved in developmental functions (Figure 4D), while this was not the case in EP156T cells (Figure 4E), suggesting that more repressed genes in PC3 cells were developmental genes.

Comparing the gene ontology of the epigenetically activated genes and repressed genes in PC3 cells versus EP156T cells, we found that both gene groups were highly associated with development and cell differentiation, suggesting that prostate carcinogenesis is a developmental related process that is involved in activating a set of developmental genes and silencing another set of developmental genes (Figure 4F). Actually, there were many known regulators in both activated and repressed groups, such as the *SOX* and *FOX* gene families in the repressed group, and cyclin-dependent kinase inhibitor 1B (*p27/Kip1/CDKN1B*) and *EZH2* in the activated group (Table S9).

### Coordinated Epigenetic Switches were Very Consistent with Gene Expression Switches of Clustered Genes

**Figure 5:**
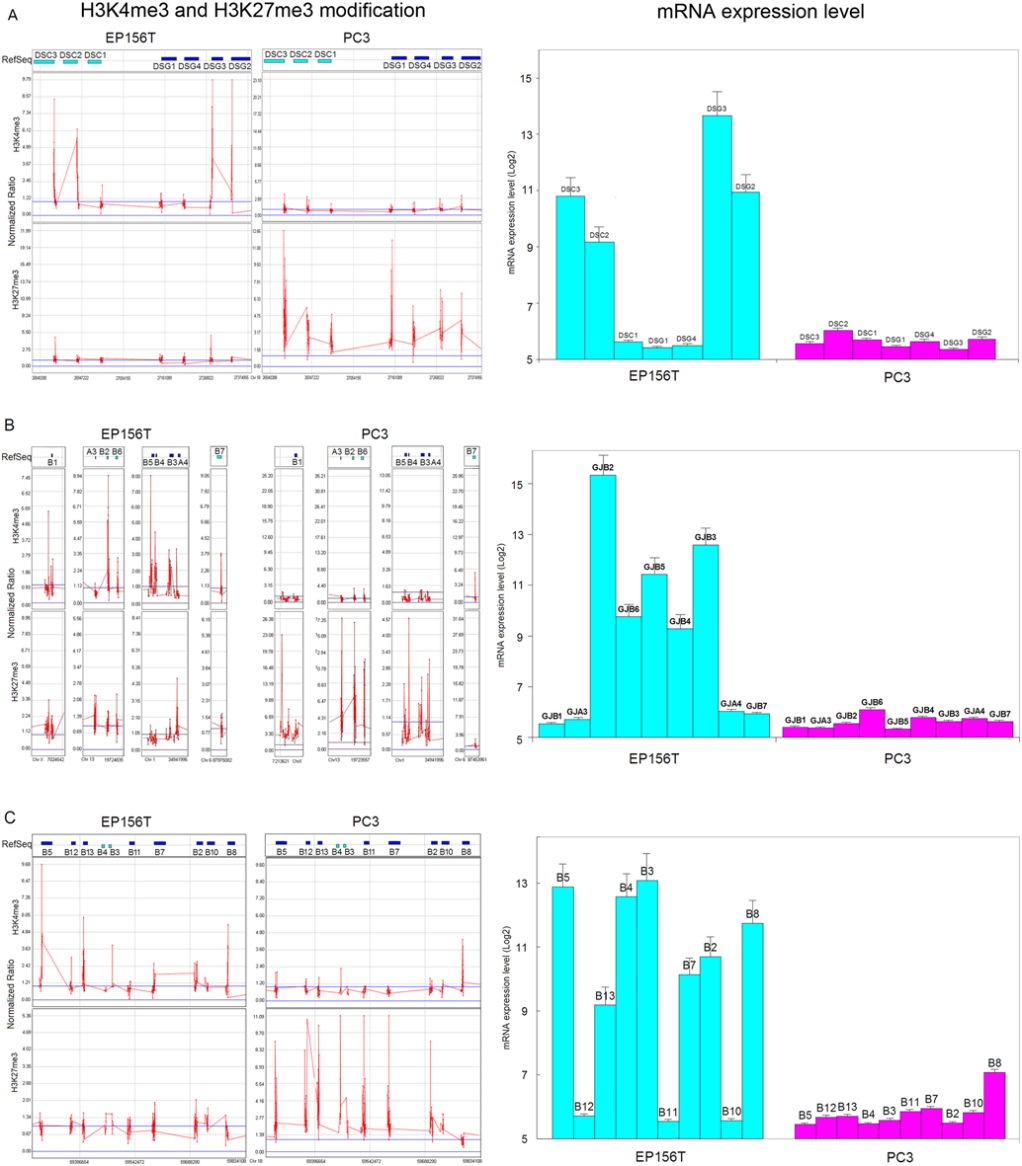
Epigenetic Switches and Decreased Expression of Gene Clusters or Gene Family. A. The *DSC* subfamily and *DSG* subfamilies were located in clusters in chr16q21. In EP156T cells, all these genes were low in H3K27me3 and four of them were high in H3K4me3, which was consistent with the high mRNA expression levels of only these high H3K4me3 marked genes. In PC3 cells, all these genes were high in H3K27me3 and low in H3K4me3 modifications, which was consistent with the low mRNA expression levels of all of them. B. Seven members of the *GJB* family are distributed among four different chromosomes: *GJB1* in ChrX, *GJB2* and *GJB6* in Chr13, *GJB3*, *4* and *5* in Chr1, *GJB7* in Chr6. In Chr13, *GJA3* was located upstream of *GJB2*, in Chr1 *GJA4* was located downstream of *GJB4*. Most of the genes contained high H3K4me3 in EP156T cells with high mRNA expression levels, while most of them contained high H3K27me3 in PC3 cells with repressed gene expression. C. There are 10 members of the *SERPINB* family localized together at 18q21. The mRNA expression values represent the average±SD of three independent triplicate microarray data.

There were gene modules of particular relevance for cancer among the activated and repressed groups. Desmosomes are intercellular junctions that tightly link adjacent cells. Downregulation of desmosomal genes is associated with epithelial to mesenchymal transition [20] and loss of desmosomal adhesion is implicated in the conversion of early stage tumors to invasive cancers [21]. The desmocollin subfamily (*DSC1*, *DSC2* and *DSC3*) and desmoglein subfamily (*DSG1*, *DSG2*, *DSG3* and *DSG4*) are main components of desmosomes and all of them are arranged in clusters in chr16q21. Very strikingly, promoters of all these members exhibited epigenetic repression (H3K27me3+ and/or H3K4me3−) in PC3 cells compared with EP156T cells (Figure 5A). Comparing gene expression data of both cell lines, we found that H3K27me3 and H3K4me3 switches are strongly associated with the expression switches of these genes from EP156T cells to PC3 cells. Plakophilin (PKP) proteins localize to cell desmosomes and nuclei, and may be involved in molecular recruitment and stabilization during desmosome formation. There are 4 genes of the *PKP* family (*PKP1–4* in Chr 1, 12, 11 and 2, respectively). All of them belonged to the repressive group and their mRNAs were downregulated in PC3 cells (Table S9, S10). Other repressed desmosome components included desmoplakin (*DSP*), an obligate component of functional desmosomes and anchors intermediate filaments to desmosomal plaques, and *PERP*, a critical component of the desmosome [22], which also exhibited high expression in EP156T cells and silencing in PC3 cells (Table S9, S10). The gap junction is another kind of cell junction that allows the transport of ions and metabolites between the cytoplasm of adjacent cells. It is formed by gap junction proteins including the alpha (GJA) and beta (GJB) families. There are seven members of the *GJB* family located in four chromosomes. We found that all members of the *GJB* family belonged to the repressed group in PC3 cells (Figure 5B). Microarray data showed that the repression of all these gap junction genes were highly consistent with their H3K4me3 and H3K27me3 modification patterns. This suggests that the H3K4me3 and H3K27me3 modifications were coordinated not only physically, but also functionally.

Serine proteinase inhibitors (SERPINs) are a superfamily with diverse functions, and are involved in a number of fundamental biological processes such as fibrinolysis, angiogenesis, programmed cell death, development and tumor suppression [23]. Genes encoding class B (*SERPINB*s) are clustered in two chromosomal regions, 18q21 and 6q25. However, all of the *SERPINB* genes that appear to be involved in cancer etiology localize together to 18q21 [24]. Based on gene expression microarray data of EP156T cells and PC3 cells, we found that all *SERPINB*s in 18q21 were silenced in PC3 cells, while 7 out of 10 were highly expressed in EP156T cells. Analysis of the H3K4me3/H3K27me3 modifications of *SERPINB*s in 18q21 in EP156T and PC3 cells showed that almost all *SERPINB*s were strongly marked with H3K27me3 only in PC3 cells, while all of them contained low H3K27me3 and 7 of them contained high H3K4me3 in EP156T cells. Very strikingly, the seven genes with high H3K4me3 corresponded exactly to the genes with high expression in EP156T cells (Figure 5C). It will be attractive to verify if carcinogenesis of prostate cancer cells were due to such coordinated epigenetic switches of these *SERPINB* genes, since some of them are known tumor suppressor genes, such as *SERPINB2* and *SERPINB5*, and can contribute to decreased tumor growth and metastasis [25], [26].

### H3K4me3 and H3K27me3 switches in HOX gene clusters in prostate cancer cells

Homeobox (HOX) transcription factors confer anterior-posterior (AP) axial coordinates to vertebrate embryos. These master regulators of development continue to be expressed throughout adulthood in various tissues and organs. Dysregulated expression of homeobox genes has been described in many solid tumors and derivative cell lines [27]. Overexpression of *HOXC4*, H*OXC5*, *HOXC6* and *HOXC8* was also reported to accompany the malignant phenotype in human prostate cells [28].

**Figure 6:**
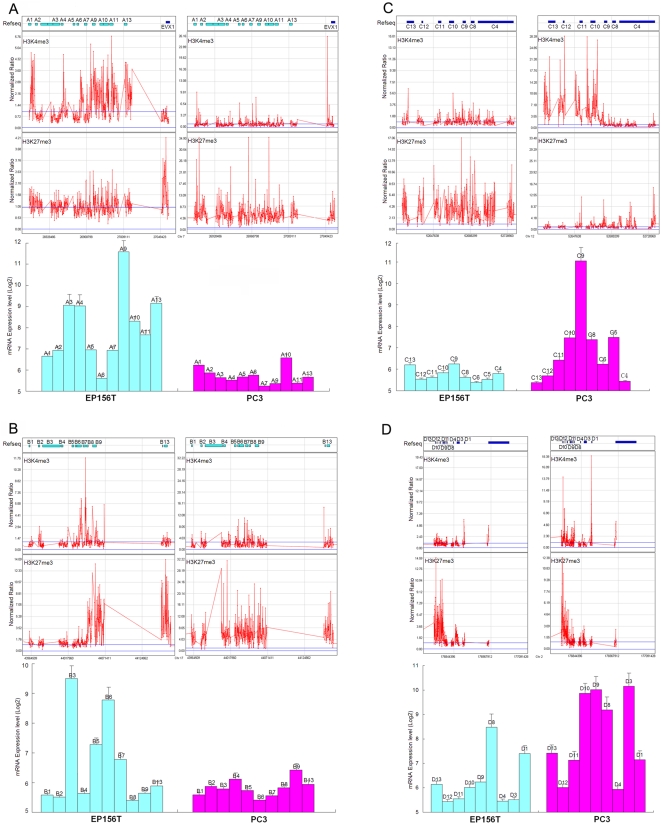
Epigenetic Switches and Expression Changes of *HOX* Cluster Genes. *HOX* genes located in 4 clusters, *HOXA* (A), *HOXB* (B), *HOXC* (C) and *HOXD* (D), in chromosome 7, 17, 12 and 2 of the human genome, respectively. The mRNA expression values represent the average±SD of three independent triplicate microarray data.

Comparing the H3K4me3 and H3K27me3 modification patterns in *HOX* gene clusters in EP156T and PC3 cells, we found that all these gene clusters were highly marked in both cells (Figure 6). Very interestingly, most of the *HOXA* genes contained high H3K4me3 and low H3K27me3 in primary prostate EP156T cells, while they contained high H3K27me3 and low H3K4me3 in prostate cancer PC3 cells (Figure 6A). In contrast, most genes in the *HOXC* clusters contained high H3K27me3/low H3K4me3 in EP156T cells and high H3K4me3/low H3K27me3 in PC3 cells. The expression levels of *HOXA* genes and *HOXC* genes in EP156T and PC3 cells showed high consistency regarding their H3K27me3/H3K4me3 patterns in both cell lines: most *HOXA* genes were much lower expressed while most of *HOXC* genes were much higher expressed in PC3 cells compared with EP156T cells (Figure 6A and B). Similar switches were also found in *HOXB* and *HOXD* clusters: most *HOXB* genes were marked with H3K4me3 alone or bivalent H3K4&K27me3 in EP156T cells, while marked by H3K27me3 alone in PC3 cells. Most of *HOXD* genes were H3K27me3 only in EP156T cells but bivalent H3K4/27me3 or H3K4me3 only in PC3 cells. The different epigenetic patterns were also consistent with their different mRNA expression levels in EP156T cells and PC3 cells (Figure 6C and D). The epigenetic switches and gene expression switches of *HOX* cluster genes in prostate cancer cells were most interesting, because *HOX* genes were identified as classic modification targets of the polycomb complex during development. All four clusters were highly enriched in H3K27me3 marks and maintained expression silencing in ES cells [13]. During development, *HOX* gene products act in a combinatorial and partly redundant manner to specify the identities of developing vertebrae [29], [30]. We found that *HOXA* and *HOXB* cluster genes were epigenetically repressed and transcriptionally silenced from normal cells to cancer cells while *HOXC* and *HOXD* cluster genes exhibited inverse switches at both epigenetic and transcriptional levels, suggesting distinct and may be inverse biological functions during prostate carcinogenesis.

### H3K4me3/H3K27me3 modification of miRNA genes in prostate cells

MicroRNAs (miRNAs) are small, endogenously expressed noncoding RNAs that regulate the expression of protein-coding genes. Increasing evidence supports miRNAs as key participants in gene regulatory networks [31]. miRNAs might function biologically as either oncogenes or tumor suppressor genes and are involved in cancer pathogenesis. A number of studies have detected frequent alterations of miRNA expression in a variety of human malignancies including prostate cancer [32], [33], [34], [35]. Epigenetic regulation of miRNA expression in cancer was addressed by treating cancer cell lines with DNA-methylation inhibitors or HDAC inhibitors [36], [37]. These data suggest that epigenetic changes play critical roles in miRNA expression in human cancers.

Using ChIP-chip analysis, we have achieved genome-wide profiles of the trimethylation of H3K4 and H3K27 in promoters of human miRNAs in both prostate primary and cancer cells. Altogether, we found that 76 miRNAs gained H3K4me3 and/or lost H3K27me3; and 114 miRNAs gained H3K27me3 and/or lost H3K4me3 in PC3 cells compared with EP156T cells, suggesting that these miRNAs were epigenetically activated and repressed in PC3 cells, respectively.

**Figure 7:**
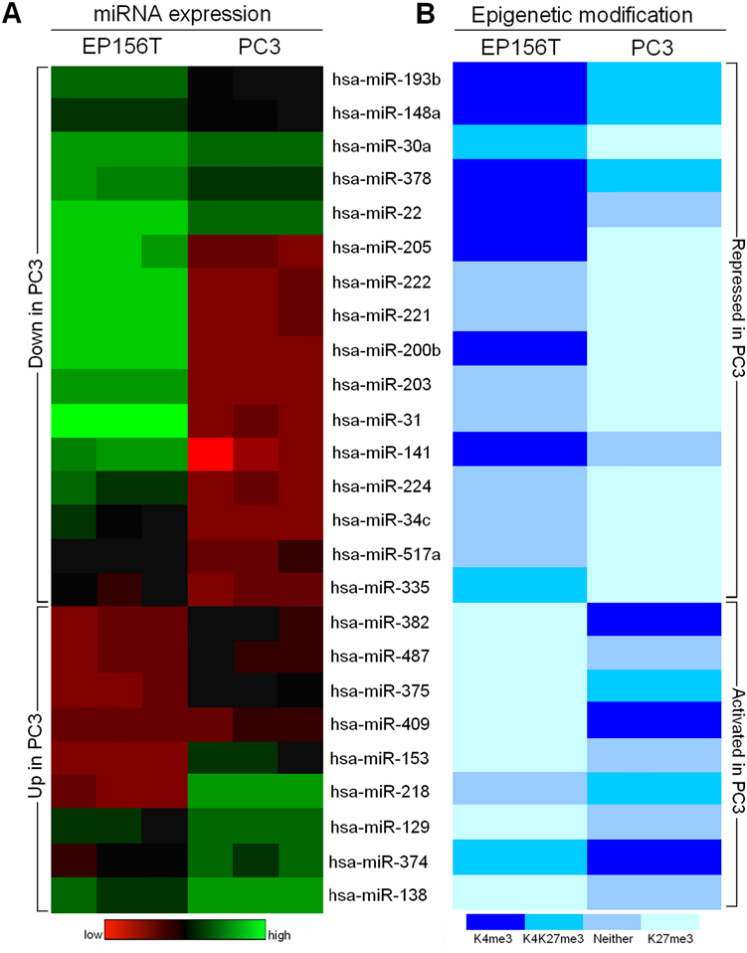
Dysregulated miRNA expression in PC3 Cells and corresponding patterns of H3K4me3 and H3K27me3 modifications. miRNA genes displayed consistent changes in both expression levels (left) and epigenetic differences (right) between EP156T and PC3 cells.

To confirm that these epigenetic switches were associated with differential miRNA transcription in prostate cells, miRNA expression microarrays were performed in both EP156T cells and PC3 cells. The comparison showed that 28 miRNAs were upregulated and 30 downregulated in PC3 cells compared to EP156T cells (Table S11, Figure 7A). Matching the lists of epigenetically activated and repressed miRNA genes according to the ChIP-chip data with the differentially expressed miRNAs in EP156T and PC3 cells, we found that up to 33% of the upregulated and 57% of the downregulated miRNAs in PC3 cells overlapped correspondingly (Figure 7A, B), thus revealing a highly consistent pattern of H3K4me3/H3K27me3 modifications coupled with miRNA expression in prostate cancer (Chi-square test, p<0.01). Among these miRNAs, some are highly relevant in cancer, such as miR-205 and miR-200b, which were H3K4me3 marked in EP156T cells and H3K27me3 marked in PC3 cells (Figure 7B), and have been reported as suppressors of the transcription factors ZEB1 and SIP1 [38], [39]. Downregulation of these miRNAs may be an important step in tumor progression. It is very likely that the epigenetic switch from H3K4me3 to H3K27me3 may be involved in decreased expression of miR-205 and miR-200b, thus increasing the metastatic ability of PC3 cells, a cell line isolated from a bone metastasis of a prostatic adenocarcinoma.

### Activated Oncogenes and Repressed Tumor Suppressor Genes in PC3 cells Indicate an H3K4me3/H3K27me3 Signature in Prostate Carcinogenesis

**Figure 8:**
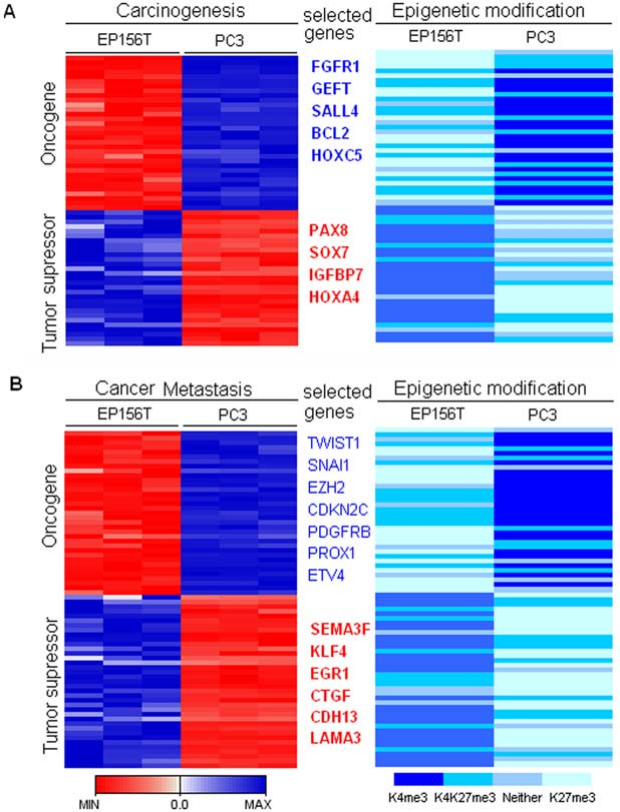
Epigenetic Signature in Prostate Cancer. A set of well defined carcinogenesis related genes (A) and metastasis related genes showed consistent epigenetic switches and gene expression changes from prostate primary (EP156T) to cancer cells (PC3).

In cancer cells, activation of oncogenes and inactivation of tumor suppressors represent critical steps at every stage of tumorigenesis. Based on the integration of epigenomic and genomic analysis of prostate cancer and primary cells, we have identified 527 upregulated genes with activated epigenetic modification and 668 downregulated genes with repressed epigenetic modification in PC3 cells. Many activated genes are known oncogenes and the repressed genes are tumor suppressor genes (Figure 8, Table S12). Oncogenes and tumor suppressor genes were separated into those previously published to play a role during carcinogenesis (Figure 8A) and those who play a role in progression of established tumors (Figure 8B). Some of these genes have known functions in prostate cancer, such as *FGFR1*, *BCL2*, *HOXC5*, *HOXA4*, *TWIST1*, *EZH2*, *KLF4*, *CTGF* (Table S12). For most of them this is the first report, to our knowledge, that their dysregulated expression in cancer may be connected to H3K4me3 and H3K27me3 switches. Our present results provide a novel view to understanding the coordinated upregulation of these oncogenes and repression of these tumor suppressor genes in carcinogenesis, thus underscoring the H3K4me3/H3K27me3 signature in prostate carcinogenesis.

## Discussion

This work has integrated microarray-based global gene expression and epigenomic profiling of prostate cancer cells and their normal counterparts and found that transcriptional activating marks (gain of H3K4me3 or loss of H3K27me3) and silencing marks (gain of H3K27me3 or loss of H3K4me3) were tightly associated with upregulated and downregulated genes in prostate cancer cells, respectively. The most prevalent switches were gain or loss of H3K27me3 coupled with the lowest enrichment of differentially expressed genes. The least prevalent switches were the ones between H3K4me3 and H3K27me3, but this was coupled with the highest enrichment of dysregulated genes, suggesting that the epigenetic marks under study are strongly linked to gene expression changes, but gain or loss of one single marker is usually not sufficient for transcriptional changes. Instead, combinations of changed epigenetic marks, which occur less frequently than changes of single marks, seem necessary in order to achieve threshold values that lead to efficient silencing or activation of genes. Furthermore, we found that mRNA expression of entire functional modules of genes is coordinated at this epigenetic level. Finally, we identified a set of overexpressed oncogenes with activated epigenetic marks and underexpressed tumor suppressor genes with repressed epigenetic marks in prostate cancer cells, indicating a strong epigenetic signature in prostate carcinogenesis. To our knowledge, this is the first report of genome-wide profiling of the activation marks H3K4me3 and the silencing mark H3K27me3 in both cancer cells and primary cells, and sheds light on the understanding of dynamic epigenetic changes during carcinogenesis.

The bivalent H3K4&K27me3 modification was first observed and proposed to be specific in ES cells [12]. Further work revealed that it was also present in differentiated cells such as T cells and MEFs [16], [17], [18]. Our data show that it also can be found in both prostate primary and cancer cells. However, the bivalent H3K4&K27me3 seems quite different between ES cells and somatic cells. Firstly, the percentage of H3K27me3 marks colocalizing with H3K4me3 was extremely high (both were 89%) in ES cells [13], [19], but considerably lower in EP156T and PC3 cells (around 50%). A similar percentage was observed in T cells (59%) [18]. Secondly, the mRNA expression of bivalently H3K4&K27me3 marked genes was very low in ES cells, with only slightly higher expression levels than H3K27me3 only marked genes [13], [17], [19]. In contrast, we found that bivalent H3K4&K27me3 marked genes were rather active and only moderately less expressed than H3K4me3 only marked genes in both EP156T and PC3 cells. Again, a similar activity of bivalent H3K4&K27me3 marked genes were observed in T cells, in which the percentages of expressed genes were 76%, 59% and 27% regarding H3K4me3 only, bivalent H3K4&K27me3 and H3K27me3 only, respectively.

Very recently, global epigenetic silencing marks in PC3 cells were reported by two groups, and both found that H3K27me3 modified loci excluded DNA hypermethylation [5], [11]. Most interestingly, the increased and decreased DNA methylation regions in *HOX* gene clusters identified by Gal-Yam *et al.* correlated very well with the switched H3K4me3/H3K27me3 regions between EP156T and PC3 cells. The decreased DNA methylation changes of the *HOXB4–B6* region, which suggested high H3K27me3 modification at these loci, exhibited high H3K27me3/low H3K4me3 in PC3 cells of our study. In the *HOXC* cluster the other study found increased DNA methylation changes, which suggested none or low H3K27me3 modification at this locus, again supporting our findings of very low H3K27me3 in the whole cluster and high H3K4me3 in the N terminal part of this cluster in PC3 cells. It will be attractive to achieve whole genome mapping of combined DNA methylation, H3K27me3 and H3K4me3 marks in both malignant and normal cells to get an even more detailed insight into epigenetic switches during carcinogenesis. Coordinated community based efforts to decode the human epigenome are planned [40]. The emerging picture is that functional groups of genes shift their gene expression patterns during carcinogenesis in association with specific combinations of epigenetic modifications. The cascade nature of epigenetic regulation may cause individual epigenetic modifications to be somewhat redundant as markers of the epigenetic state, and this might facilitate an overall understanding of coordinated regulation of gene expression in cancer and facilitate identification of molecular targets of cancer therapy.

## Materials and Methods

### Cell lines, Antibodies and Reagents

The PC3 prostate cancer cell line was obtained from the American Type Culture Collection (ATCC, Rockwell, MD, USA). The EP156T primary prostate epithelial cells have been described [10]. Antibodies against H3K4me3 and H3K27me3 were bought from Abcam. Reagents for real-time qPCR were from Applied Biosystems, Foster City, USA.

### Global Gene Expression Analysis

The Agilent Human Whole Genome (4×44 k) Oligo Microarray with Sure Print Technology (Agilent Technologies, Palo Alto, CA, USA) was used to analyze samples in the present study. Quality and yields of total RNA were assessed using the Agilent 2100 Bioanalyzer (Agilent Tech), 1% agarose gel ethidium bromide electrophoresis and Powerwave spectrophotometry at 260 nm and 280 nm. One µg of DNAse-treated total RNA was converted into cDNA and Cy3-labeled cRNA using the Low RNA Input Linear Amplification Kit PLUS, One-Color kit (Agilent Tech.) according to instructions. We used gmeansignals, *i.e.* signals without background subtraction. Intraarray normalization of dye effects was carried out using quantile normalization [42] and genes with more than 25% missing values were removed. The normalized channel values were log(2) transformed and combined into a gene expression data matrix. Data were formatted in a J-Express-file suitable for additional data mining (http://www.molmine.com/) [43]). Following normalisation, analysis of variance (ANOVA) of the J-Express program package was used for identification of differentially expressed genes. Only genes that changed more than 1.2 fold and with p-values<0.005 were considered as differentially expressed genes between the prostate benign and cancer groups, and only genes that changed more than 2.0 fold with p-values<0.01 were considered as differentially expressed genes in cell lines.

### miRNA Microarray Analysis

The global miRNA expression of each sample was measured in triplicates using the Febit Genome One technology (Febit Biomed GmbH, Heidelberg, Germany). Each microarray contains the reverse complement of 866 miRNAs and mature star sequences as published in the Sanger mirBase 12.0. Seven replicates of these probes were synthesized in random order on the array together with control probes. Total RNA was purified using the miRNeasy Mini kit (Qiagen), the quality control was done with the Agilent Bioanalyzer 2100, using the RNA 6000 nano kit (Agilent). For each array 1.5 µg total RNA was labeled using the mirVANA™ miRNA labeling kit (Ambion) following the manufacturer's instructions. Then, samples were hybridized for 16 hours at 42°C on the biochip. During the incubation, samples were slightly moved using Argon pressure. The washing and signal amplification passed according to Febit's standard procedures and criteria. Signal intensities were measured by autoexposure time using a CCD camera. Raw data were further processed by the statistical programming framework R: A background correction was done. The intraarray replicates were merged by computing their mean intensity. Standard quantile normalization was applied to account for interarray effects. Finally, probes that did not show expression (normalized intensity below 500) were removed. To detect probes with significant differential expression, moderate t-statistics were calculated and the respective p-values were adjusted for multiple testing using Benjamin-Hochberg correction.

### Chromatin Immunoprecipitation on DNA Microarrays (ChIP-chip)

ChIP-chip was performed according to the Agilent ChIP-chip protocol with modifications. To immunoprecipitate chromatin, 6×10^7^ cells were treated with 1% formaldehyde at room temperature for 10 min followed by quenching with 0.125 M glycine. Cells were lysed and the nuclei were sonicated under conditions yielding fragments ranging from 200 bp to 800 bp. Five per cent of the sonicated material was saved as whole-cell extract. Sonicated lysate was divided to three equal volumes and immunoprecipitated with specific or negative antibody bound to protein A magnetic beads (Invitrogen) overnight at 4°C with rocking. Antibodies used were against: H3K4me3 (Abcam no. ab8580) or H3K27me3 (Abcam no. ab6002) or mouse IgG (Sigma). Five µg of antibody was used per 2×10^7^cells. Immunoprecipitated complexes were collected, washed and eluted using Dynal Magnetic Particle Concentrator (Invitrogen). Eluted DNA and whole-cell extracts were incubated at 65°C in a rotating incubator for 8 hours to reverse crosslinks. DNA samples were sequentially treated with RNase A and proteinase K and then purified by phenol/chloroform extraction. The purified DNA was ethanol precipitated using glycogen as a carrier and resuspended in PCR-grade water. For each microarray, 2 µg ChIPed DNA was labeled using the CGH kit (Invitrogen) and Cy3-dUTP or Cy5-dUTP (Amersham). Human G4489A 2×244 K promoter arrays (Agilent) were hybridized for 40 h at 65°C and subsequently scanned using an Agilent Scanner controlled by Agilent Scan Control 7.0 software. Raw image files were extracted with Agilent Feature Extraction 9.1 software. H3K4me3 or H3K27me3 enriched genes were analyzed using Agilent's ChIP Analytics 1.3 software, incorporating the Whitehead Error Model, which has a false-positive rate of approximately 0.5% and a false-negative rate of approximately 20%. Probes that were associated with modified histones were called on the basis of a neighbourhood p-value of less than 0.05. Mapping of bound probes was performed using human genome (HG17, May 2004).

### ChIP-PCR

Real-time quantitative PCR was done to verify the ChIP-chip data. Taqman primers (Table S13) were designed to detect the promoter region (−5.5 Kb upstream and +2.5 Kb downstream of TSS). 10 ng ChIPed DNA and input DNA were used for amplification, the enrichment of ChIPed DNA was calculated based on the ΔCt value, 3.5 ΔCt equals 10 fold of the amount of target DNA. Three biological and technical replicates were done for each sample.

### BASE database, ArrayExpress and Microarray Accession Numbers

Annotated microarray data were uploaded in the BASE database (http://www.cbu.uib.no), formatted and exported to ArrayExpress at the European Bioinformatics Institute (http://www.ebi.ac.uk/microarray/) according to MIAME guidelines (Gene Expression study ArrayExpress accession number: E-TABM-634; Histone Methylation pattern ChIP-chip study ArrayExpress accession number: E-TABM-635).

### Gene Ontology Analysis

Gene ontology was performed using DAVID Bioinformatics Resources (http://david.abcc.ncifcrf.gov/).

## Supporting Information

Figure S1 ChIP-qPCR Examination of H3K4me3 and H3K27me3 Modifications in EP156T and PC3 Cells
(0.05 MB DOC)

Table S1 Epigenetic genes changed in prostate cancer and benign tissues
(0.03 MB XLS)

Table S2 H3K4me3 marks in EP156T cells
(10.09 MB XLS)

Table S3 H3K27me3 marks in EP156T cells
(8.36 MB XLS)

Table S4 H3K4me3 marks in PC3 cells
(7.12 MB XLS)

Table S5 H3K27me3 marks in PC3 cells
(8.96 MB XLS)

Table S6 Genes with H3K4me3, H3K4K27me3, H3K27me3 and Neither marks in X chromosome
(0.05 MB XLS)

Table S7 Genes with H3K4me3, H3K4K27me3, H3K27me3 and Neither marks in EP156T cells
(0.84 MB XLS)

Table S8 Genes with H3K4me3, H3K4K27me3, H3K27me3 and Neither marks in PC3 cells
(1.14 MB XLS)

Table S9 Epigenetic activated and repressed genes in PC3 cells
(0.48 MB XLS)

Table S10 Differentially expressed genes in EP156T and PC3 cells
(0.74 MB XLS)

Table S11 Differentially expressed miRNAs in EP156T cells and PC3 cells
(0.03 MB XLS)

Table S12 Epigenetic activated oncogene and repressed tumor suppressor genes
(0.09 MB XLS)

Table S13 Oligo sequences of Taqman probes for ChIP-PCR
(0.02 MB XLS)
