# Supplementary material for: Genome-Wide Profiling of Histone H3 Lysine 4 and Lysine 27 Trimethylation Reveals an Epigenetic Signature in Prostate Carcinogenesis
Source: PLoS One. 2009 Mar 5;4(3):e4687. doi: 10.1371/journal.pone.0004687 (PMC2650415; doi:10.1371/journal.pone.0004687)
Supplement: Figure S1 — ChIP-qPCR Examination of H3K4me3 and H3K27me3 Modifications in EP156T and PC3 Cells (0.05 MB DOC) [file pone.0004687.s001.doc]

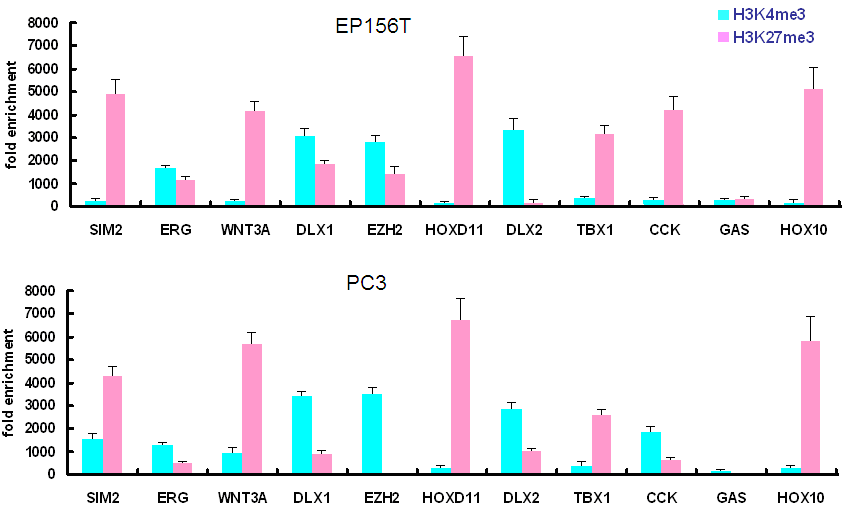


**Figure S1. ChIP-qPCR Examination of H3K4me3 and H3K27me3 Modifications in EP156T and PC3 Cells**

Enrichment of promoters of 11 selected genes following chromatin immunoprecipitation (ChIP) using ChIP-grade anti-H3K4me3 and anti-H3K27me3 antibodies in EP156T (above) and PC3 (below) cells as determined by quantitative PCR amplification. Fold Enrichment was calculated based on the ΔCt values between the ChIPed DNA and input DNA (see Experimental Procedures). All the results represent the median of three independent triplicate experiments.

.
